# The extent and risk of knee injuries in children aged 9–14 with Generalised Joint Hypermobility and knee joint hypermobility - the CHAMPS-study Denmark

**DOI:** 10.1186/s12891-015-0611-5

**Published:** 2015-06-12

**Authors:** Tina Junge, Lisbeth Runge Larsen, Birgit Juul-Kristensen, Niels Wedderkopp

**Affiliations:** Institute of Regional Health Research, University of Southern Denmark, Odense, Denmark; Department of Physiotherapy, University College Lillebaelt, Odense, Denmark; Health Sciences Research Centre, University College Lillebaelt, Odense, Denmark; Centre for Welfare Technology Research and Development, University College Lillebaelt, Odense, Denmark; Department of Sports Science and Clinical Biomechanics, University of Southern Denmark, Odense, Denmark; Institute of Occupational Therapy, Physiotherapy and Radiography, Bergen University College, Bergen, Norway; Spine Centre of Southern Denmark, Hospital Lillebaelt, Middelfart, Denmark; IRS, SDU, Winsløwparken 19,3. 5000, Odense C, Denmark

**Keywords:** Knee injuries, Generalised Joint Hypermobility, Children, Beighton tests

## Abstract

**Background:**

Generalised Joint Hypermobility (GJH) is suggested as an aetiological factor for knee injuries in adolescents and adults. It is presumed that GJH causes decreased joint stability, thereby increasing the risk of knee injuries during challenging situations like jumping and landing. The aim was to study the extent and risk of knee injuries in children with GJH and knee hypermobility.

**Methods:**

In total, 999 children (9–14 years) were tested twice during spring 2012 and 2013 with Beighton´s Tests (BT) for hypermobility, a 0–9 scoring system. GJH was classified with cut-point ≥5/9 on both test rounds. On basis of weekly cell phone surveys of knee pain, children requiring clinical examination were seen. Traumatic and overuse knee injuries were registered by WHO ICD-10 diagnoses. Logistic regression and Poisson regression models with robust standard errors were used to examine the association between GJH and knee injuries, taking into account clustering on school class levels.

**Results:**

Totally, 36 children were classified GJH on both test rounds. Overuse knee injuries were the most frequent injury type (86 %), mainly apophysitis for both groups (61 %), other than patella-femoral pain syndrome for the control group (13 %). For traumatic knee injuries, distortions and contusions were most frequent in both groups (51 % resp. 36 %), besides traumatic lesions of knee tendons and muscles for the control group (5 %). No significant association was found between overuse knee injuries and GJH with/without knee hypermobility (OR 0.69, *p* = 0.407 resp. OR 0.75, *p* = 0.576) or traumatic knee injuries and GJH with/without knee hypermobility (OR 1.56, *p* = 0.495 resp. OR 2.22, *p* = 0.231).

**Conclusions:**

Apophysitis, distortions and contusions were the most frequent knee injuries. Despite the relatively large study, the number of children with GJH and knee injuries was low, with no significant increased risk for knee injuries for this group. This questions whether GJH is a clinically relevant risk factor for knee injuries in school children aged 9–14 years. A fluctuation in the individual child´s status of GJH between test rounds was observed, suggesting that inter- and intra-tester reproducibility of BT as well as growth may be considered important confounders to future studies of children with GJH.

## Background

In children and adolescents, the knee is one of the most frequent sites for both traumatic and overuse injuries [1–3]. In Denmark, 25 % of all children and adolescents (10–19 years) are treated each year in emergency departments because of sports-related injuries [4]. This number typically reflects traumatic injuries, since overuse injuries are often not registered in emergency departments [5, 6]. A traumatic injury is defined as one resulting from a specific, identifiable event, whereas an overuse injury is caused by repeated micro trauma without a single, recognisable event responsible for that injury [7].

Knee injuries have multifactorial origins [8, 9] including biomechanical causes such as increased joint mobility, as seen in individuals with Generalised Joint Hypermobility (GJH) [10–14]. Joint hypermobility is a variation of normal joint mobility, with GJH defined as an increase in mean joint range of motion [15]. The prevalence of GJH in children varies from 7-29 %; the large variation is likely due to heterogeneity of the studied population regarding age, sex and/or a variation in test procedures, interpretation of results and criteria used [16–18]. GJH is most often classified by the Beighton Tests (BT) for hypermobility, a 0–9 scoring system [19]. In both children and adult populations, the reproducibility of the BT criteria with cut-point ≥5/9 is moderate to substantial, with a related overall agreement of 80-88 % [20, 21]. In several studies, it is suggested that the BT be included as a predictive screening tool for knee injuries [22–24].

To protect against knee injuries, knee joint stabilisation is provided by active (neuromuscular) and passive (joint capsules and ligaments) components [22]. GJH implies decreased stiffness of the passive components [25], hypothetically increasing the risk of knee injuries during challenging situations requiring a high level of knee joint stabilisation, such as jumping, landing and pivoting. In support of this notion, a positive association between GJH and knee injuries was reported in a recent meta-analysis, where sport participants (9–39 years) with GJH at BT cut-point ≥4/9 had five times the risk of knee injuries, especially during contact sport activities [14]. Also, Anterior Cruciate Ligament (ACL) injuries were more frequent in adults with GJH at BT cut-point ≥6/9 and knee hypermobility in the contralateral, uninjured knee in a case–control study of patients undergoing an ACL reconstruction [26]. In contrast, no such associations were found between knee injuries and GJH or knee joint laxity, measured as increased anterior-posterior tibio-femoral translation, in a prospective cohort study of female, adult soccer players at BT cut-point ≥4/9 [27].

In children and adolescents, an increased risk of knee injuries for individuals with GJH was seen in specific sport populations. In a cross-sectional study of junior netball players (6–16 years), the odds of sustaining an injury, primarily in the ankles and knees, were three times higher for the GJH group at BT cut-points 5-9/9 [10]. For female soccer and basketball players (14–19 years) the odds of ACL injuries was five times greater for those with knee hypermobility and knee joint laxity in a prospective case–control study [22].

In summary, knowledge of an association between GJH and knee injuries is mainly derived from studies of adults and in sports-specific studies, while knowledge of the influence of GJH on knee injuries in children and adolescents is sparse. Moreover, most studies report only prevalence of traumatic injuries [14], meaning that there is a lack of knowledge about overuse injuries. For dose–response calculations, data on both traumatic and overuse injuries measured in longitudinal studies are essential [28].

Therefore, the aims of this study were to evaluate the extent of knee injuries in children and adolescents with GJH and knee hypermobility and to examine the risk of knee injuries for the GJH group when compared with a control group in a longitudinal cohort study.

## Methods

### Design

This study was nested in The Childhood Health, Activity and Motor Performance School Study Denmark (the CHAMPS-Study DK), a longitudinal cohort study launched in 2008, following children from 10 public schools in the Municipality of Svendborg, Denmark [29]. Six schools volunteered as sports schools with six physical education lessons per week and four normal schools served as controls with two physical education lessons per week. Data for the current study involved the period March 2012 to June 2013 from all schools.

The Regional Scientific Ethics Committee for Southern Denmark approved the experimental protocol (jnr. S-20080047 HJD/csf) and the study was reported to the Danish Data Protection Agency. Written and oral information about participation in the study was provided to the parents or guardians of each child according to the Declaration of Helsinki [30]. Written informed consent for participation was received, and all participation was voluntary with the option to withdraw from the project at any time. Prior to every clinical examination for musculoskeletal injuries, an additional verbal agreement was obtained from each child and his/her respective parents.

### Participants

**Fig. 1:**
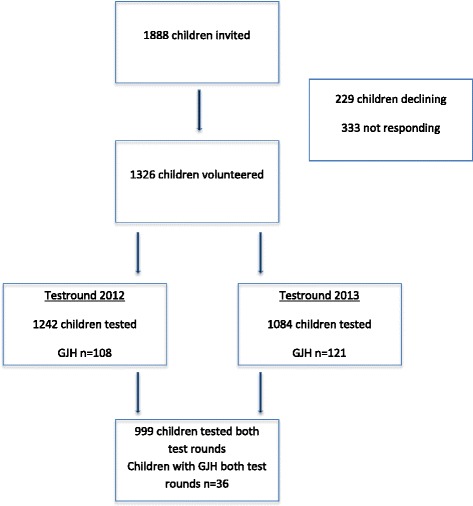
Flow chart of children enrolled in the study

In total, 1888 children and adolescents from the third to the eighth grades, from 10 public schools, were invited to participate in the longitudinal registration of musculoskeletal injuries (Fig. 1). The age span in this study was 9–14 years. The study was kept open, with the possibility for new children to enter.

Exclusion criteria for the current study were children with diagnosis of a chronic musculoskeletal or neurological condition, or pain in the regions being examined in the BT on the day of testing.

### Outcome measures

#### Clinical tests and anthropometry

The children and adolescents participating in the study were clinically examined on two test rounds (spring 2012 and spring 2013) using BT and criteria for GJH [19, 20] (Fig. 1). BT consists of five manoeuvres: 1) passive dorsiflexion of the little fingers beyond 90°, 2) passive apposition of the thumbs to the flexor aspects of the forearm, 3) hyperextension of the elbows beyond 10°, 4) hyperextension of the knees beyond 10° and 5) forward flexion of the trunk with the knees straight, resting the palms easily on the floor [19]. One point was allocated for each of the tests being positive as described, bilaterally for manoeuvres 1–4, with a total score ranging from 0–9. Cut-points ≥5/9 and ≥6/9 and ≥7/9 were intended for the analyses. The children were categorised as GJH or controls according to the described cut-point. The child and the parents were not informed about the status of GJH.

The children were tested with BT in the mornings and did not attend any physical education classes, nor perform warm-up exercises or stretching before the BT. The children were tested with BT in a random order by a team of 25 physiotherapy students on each test round. All testers were trained by two experienced physiotherapists (TJ & LR) in a standardised protocol for the BT, describing the test procedures in detail [20].

Anthropometric measures in the form of height and weight were collected simultaneously with the BT.

#### SMS-surveys

##### A) Knee injuries

Registration of knee injuries was performed in two steps:

1. To avoid recall bias, data collection was undertaken at short intervals. A Short Message Service (SMS) survey as a method for injury registration has been shown to be satisfactory for capturing both severe and less severe, traumatic, and overuse injuries, with nearly two-thirds of the injuries in children 6–10 years found to be overuse injuries [31]. Every Sunday, except for the summer holidays and Christmas holidays, the children and their parents received an SMS, asking “Has your child had any pain during the past week”? The possible answer options were one of four numbers, corresponding to pain or complaints located in 1) the back, 2) the arms, 3) the legs or 4) no pain. Every week, complete lists of those children with a positive answer to 1), 2) and/or 3) were extracted from the database, and the parents were contacted via telephone by physiotherapists and chiropractors from the CHAMPS-Study DK to determine the need for a clinical examination.
2. The need for clinical examination was based on the severity, the character and the extent of the child´s musculoskeletal pain or complaint, as described by the parents. Continued pain always merited a clinical examination. The children with a need for clinical examination were examined at their respective schools every week or fortnight by physiotherapists or chiropractors blinded to the status of GJH during the test rounds. The traumatic and overuse injuries were classified according to the ICD-10 by WHO [32], hereby expressing the severity of injury. If needed, the child was referred for further para-clinical examination, such as X-ray, ultrasound or magnetic resonance imaging scan (MRI). To get a complete data collection on injuries, information of children being diagnosed elsewhere (e.g. hospital emergency department) during the study period was collected concurrently.

##### B) Organised sports activity

The weekly amount of organised sports activity, reported by the parents to each child as the number of times spent in organised sport activity, was registered by the SMS survey every Sunday. The question was: “How many times did your child participate in organised leisure time sport within the last week?” with the possibility of answering the relevant number between 0 and 8, with 8 meaning more than 7 times. The weekly amount was expressed in times, which is not equivalent to hours for all sport types; therefore, the term ´sport participation units´ is used throughout the manuscript.

### Statistical analyses

The Students’ unpaired t-test was used to compare the characteristics of children with GJH and controls. GJH was intended to be analysed at three different cut-points: ≥5/9, ≥6/9 and ≥7/9, but due to the low prevalence of children with GJH at cut-points ≥6/9 and ≥7/9 with injuries, only cut-point ≥5/9 was used for presentation and analyses.

The following groups were defined for the analyses:

1)

- Children with a constant status of GJH, meaning that the child had a BT score of ≥5/9 on both tests rounds (n = 36).
- A further stratification of this group (1a) into a subgroup having simultaneous knee hypermobility of at least one knee during at least one of the test rounds (n = 26).

Due to an observed fluctuation of the status of GJH between test rounds (for example, a child could be classified control on the first test round with a BT score of 4, while on the second test round the BT score could be 5, classifying the child as GJH), further subgroup analyses were performed. As a measurement error of 12 % was seen in previous studies for the BT score (overall agreement 88 %)[20, 21], scores from the children classified with GJH were allowed to fluctuate one point in the BT score (from 4 in 2012 to 5 in 2013 and vice versa). Therefore, post hoc analyses on the following groups were calculated for:

2)

- Children with a constant status of GJH, as described in 1a, but including children fluctuating one point in BT between test rounds (n = 119)
- This group (2a) was further stratified into a subgroup having simultaneous knee hypermobility of at least one knee during at least one of the test rounds (n = 56).

The type of knee injury, both traumatic and overuse, was registered as count data, containing information of the total number of injuries for each child. Due to a relatively low number of injuries for the GJH group, data for both traumatic and overuse injuries were also converted to a dichotomy score of having an injury or not for each child, in order to perform the appropriate analyses. The diagnoses, expressing the severity of the injuries, were used for the descriptive reporting.

For each child, the mean weekly participation units in organised sports activity during the study period was calculated, and further used at a group level for analyses. Growth was calculated as the delta value between the two test rounds and introduced as a confounder in all analyses.

A logistic regression model (for analysis of group 1a and 1b) and Poisson regression model (for analysis of group 2a and 2b) with robust standard errors were used to test the associations between GJH and knee injuries, taking into account clustering on school class levels. All analyses were adjusted for sex, age, school type, sports participation and growth, with growth expressed as a delta value for the increase in height between the two test rounds. Only children participating in the SMS survey, answering at least 80 % of the SMS questions were included in the final analyses. The results are presented as Odds Ratios (OR) and Incidence Rate Ratios (IRR).

All statistical analyses were performed using STATA (version 13.0: Statacorp, College Station, Texas, USA) with the pre-specified level of significance being 0.05.

## Results

At baseline, 1326 children (70 %) volunteered to participate in the current study, with 229 (12 %) children and their parents declining to participate, and 333 (18 %) children and their parents not responding. The response rate for the SMS survey was 97 % for the entire period of 63 weeks, excluding periods of summer (6 weeks) and Christmas holiday (1 week). For BT and anthropometry performed during spring 2012, 94 % (n = 1242) of the children were examined, and for the tests during spring 2013, 82 % (n = 1084) were examined. In total, 999 children were tested on both rounds.

### Growth

Boys aged 13 had the largest increase in height between test rounds (mean 8 cm, maximum 15 cm). For girls, the 11 year-olds had the largest increase in height (mean 7 cm, maximum 13 cm). The growth for children with GJH is presented in Table 1.

**Table 1:** **Demography of children with four definitions of GJH for test rounds 2012 and 2013**

	Children with GJH on both test rounds (group 1a, n = 36)	Children with GJH and knee hypermobility on both test rounds (group 1b, n = 26)	Children with GJH in both test rounds with a fluctuation of 1 point in BT score (group 2a, n = 119)	Children with GJH on both test rounds with a fluctuation of 1 point in BT score and knee hypermobility (group 2b, n = 56)
**Participants (no. boys/girls)**	7/29	6/20	40/67	17/31
**Age (yrs)**				
Test round 1	10.7 ± 1.3	10.7 ± 1.3	10.9 ± 1.4	11.13 ± 1.4
**Height (cm)**				
Test round 1	148.7 ± 10.6	148.7 ± 11.7	149.9 ± 10.1	150.9 ± 11.5
Test round 2	154.5 ± 10.2	154.3 ± 11.3	155.5 ± 9.7	156.5 ± 10.6
**Growth**				
Test round 2 - Test round 1	5.8 ± 1.8	5.5 ± 1.7	5.6 ± 1.8	5.5 ± 1.8
**Body mass (kg)**				
Test round 1	38.5 ± 8.9	38.7 ± 9.7	40.8 ± 10.3	42.2 ± 12.2
Test round 2	42.8 ± 10.3	42.7 ± 11.2	43.8 ± 10.1	44.7 ± 10.5
**BMI (kg/m** ^**2**^ **)**				
Test round 1	17.2 ± 2.2	17.2 ± 2.3	17.9 ± 2.7	17.2 ± 2.3
Test round 2	17.6 ± 2.7	17.7 ± 2.9	17.8 ± 2.5	18.1 ± 2.6
**School type**				
(no. control/intervention)	9/27	6/20	46/73	21/35
Sports participation units (times/week)	1.6 ± 1.2	1.6 ± 1.2	1.4 ± 1.1	1.6 ± 1.2

GJH = Generalised Joint Hypermobility, Growth delta value of height between test rounds 2012 and 2013, BMI = Body Mass Index. Values are the mean ± SD unless otherwise indicated

### Status of GJH

There was no significant difference in demographics between groups of children with GJH and controls for the test rounds of 2012 and 2013 (Table 2). The prevalence of GJH across ages in test round 2012 was 9 % and 11 % in test round 2013 (Table 2). The gender difference increased by age, indicating the prevalence of GJH to increase for girls and to decrease for boys (not shown in tables).

**Table 2:** **Demography of children with GJH and controls for the test rounds in 2012 and 2013**

	2012		2013	
	GJH ≥5/9 (n = 108)	Controls (n = 1135)	GJH ≥5/9 (n = 121)	Controls (n = 992)
Participants (no. boys/girls)	40/68	565/569	33/88	502/490
Age (yrs)	11.0 ± 1.3	11.2 ± 1.4	11.9 ± 1.4	12.2 ± 1.3*
Height (cm)	150.4 ± 10.3	152.1 ± 10.8	156.3 ± 10.3	158.1 ± 10.9
Body mass (kg)	40.7 ± 9.7	42.4 ± 10.4	45.8 ± 10.9	47.0 ± 10.9
BMI (kg/m^2^)	17.7 ± 2.4	18.1 ± 2.6	18.5 ± 2.9	18.5 ± 2.6
School type (no. control/intervention)	43/65	461/673	38/83	362/630
Sports participation units (times/week)	1.8 ± 1.2	1.7 ± 1.2	1.7 ± 1.2	1.7 ± 1.2

GJH = Generalised Joint Hypermobility, BMI = Body Mass Index

Values are the mean ± SD unless otherwise indicated

*Significant difference between groups (*p* = 0.03 for age)

Approximately one third of the children classified with GJH had a constant status of GJH on both test rounds, corresponding to 36 children (3 %)(group 1a), and of these, 26 children (2 %)(group 1b) had simultaneous knee hypermobility of at least one knee during at least one test round. In both groups, girls were mostly represented, by 81 % (n = 29) in group 1a and 77 % (n = 20) in group 1b.

Allowing a fluctuation of one point in the BT score between test rounds resulted in 119 (12 %) children with a constant status of GJH (group 2a), with 56 (47 %) of these having simultaneous knee hypermobility of at least one knee during at least one test round (group 2b). Girls represented 63 % (n = 75) in group 2a, and 65 % (n = 36) in group 2b.

### Extent of injuries

For the total group (both GJH and controls), 610 knee injuries, hereby 14 % (n = 85) traumatic injuries (range 1–4) in 77 children and 86 % (n = 525) overuse injuries (range 1–4) in 500 children were registered during the entire period.

For the group with a constant status of GJH (n = 36, group 1a), 15 knee injuries in total were recorded, with one traumatic injury for about every three overuse injuries registered. For the group with GJH and simultaneous knee hypermobility, one traumatic injury for every two overuse injuries was seen (n = 26, group 1b) (Table 3).

**Table 3:** **Total number, traumatic and overuse knee injuries for four definitions of children with GJH and controls**

	Total number of knee injuries for controls (% of injuries in the population)	Total number of knee injuries for GJH (% of injuries in the population)	Traumatic knee injuries (% traumatic injuries in GJH)	Overuse knee injuries (% overuse injuries in GJH)
Children with GJH on both test rounds (group 1a, n = 36)	595 (97 %)	15 (3 %)	4 (27 %)	11 (73 %)
Children with GJH and knee hypermobility on both test rounds (group 1b, n = 26)	597 (98 %)	13 (2 %)	4 (31 %)	9 (69 %)
Children with GJH in both test rounds with a fluctuation of 1 point in BT score (group 2a, n = 119)	568 (93 %)	42 (7 %)	8 (19 %)	34 (81 %)
Children with GJH on both test rounds with a fluctuation of 1 point in BT score and knee hypermobility (group 2b, n = 56)	584 (96 %)	26 (4 %)	5 (19 %)	21 (81 %)

GJH = Generalised Joint Hypermobility. Group 1a) Children with a constant status of GJH, 1b) Children with a constant status of GJH and simultaneous knee hypermobility of at least one knee during at least one of the test rounds, 2a) Children as described in 1a, but including children fluctuating with one point in the BT score between test rounds, 2b) Children as described in 2a and simultaneous knee hypermobility of at least one knee during at least one of the test rounds. Values presented as absolute numbers with relative numbers in brackets

Including the group of children with a fluctuation of one point in the BT score between test rounds more than doubled the number of injuries for the GJH group, mainly due to an increase in the number of overuse injuries (Table 3).

The traumatic knee injuries seen in both groups consisted of distortions (51 %, n = 43) and contusions (36 %, n = 31), other than traumatic lesions of tendons and muscles of the knee (5 %, n = 4) in the control group.

For overuse injuries, the most frequent diagnosis in both groups was Mb. Sinding-Larsen-Johansson (38 %, n = 200) and Mb. Osgood-Schlatter (23 %, n = 123), other than patella-femoral pain syndrome (13 %, n = 69) in the control group.

### Associations

No significant association was found between GJH and the total amount or type of knee injuries, whether traumatic or overuse knee injuries (Tables 4 and 5).

**Table 4:** **Odds Ratio for knee injuries in children with GJH with/without knee hypermobility**

	OR (95 % CI)	*P* value	OR (95 % CI) unadjusted values	*P* value
**GJH, group 1a (n = 36)**				
All injuries	0.83 a (0.37 - 1.84)	0.649	0.93 (0.49-1.78)	0.840
Traumatic injuries	1.56 c (0.43 - 5.61)	0.495	1.59 (0.66-3.86)	0.297
Overuse injuries	0.69 b (0.29 - 1.65)	0.407	0.73 (0.37-1.45)	0.379
**GJH and knee hypermobility, group 1b (n = 26)**				
All injuries	0.99 a (0.40 - 2.44)	0.981	1.24 (0.60-2.55)	0.553
Traumatic injuries	2.22 c (0.60 - 8.19)	0.231	2.34 (0.94-5.80)	0.066
Overuse injuries	0.75 b (0.27 - 2.06)	0.576	0.89 (0.42-1.92)	0.782

Values are Odds Ratios (OR) adjusted for sex, age, school type, sports participation and growth with 95 % confidence intervals. Logistic regression model. *P* value indicates main effect of GJH on knee injuries. GJH = Generalised Joint Hypermobility. Groups presented by children with GJH (group 1a) and children with GJH and simultaneous knee hypermobility (group 1b). Unadjusted values represent the association of injury types and GJH only

a = sex, age and sports participation significance, growth borderline significance

b = sex, age, growth and sports participation significance

c = sports participation significance, growth borderline significance

**Table 5:** **Incidence Rate Ratio of knee injuries in children with GJH with a fluctuation of ±1 point**

	IRR (95 % CI)	*P* value	IRR (95 % CI) unadjusted values	*P* value
**GJH ±1 point, group 2a (n = 119)**				
All injuries	0.84 a (0.48 - 1.49)	0.565	0.76 (0.60-0.96)	0.026
Traumatic injuries	1.45 c (0.40 – 5.21)	0.567	1.08 (0.64-1.81)	0.757
Overuse injuries	0.76 b (0.40 - 1.44)	0.404	0.70 (0.54-0.92)	0.010
**GJH ±1 point and knee hypermobility, group 2b (n = 56)**				
All injuries	1.10 a (0.61 - 1.94)	0.750	1.09 (0.82-1.44)	0.538
Traumatic injuries	2.18 c (0.63 – 7.52)	0.215	1.35 (0.71-2.56)	0.350
Overuse injuries	0.94 b (0.48 - 1.81)	0.858	1.04 (0.76-1.42)	0.789

Values presented as Incidence Rate Ratios (IRR) adjusted for sex, age, school type, sports participation and growth with 95 % confidence intervals. Poisson regression model. *P* value indicates main effect of GJH on knee injuries. GJH = Generalised Joint Hypermobility. Groups presented by children with GJH and a fluctuation of ±1 point in the BT score (group 2a) and children with GJH with a fluctuation of ±1 point and simultaneous knee hypermobility in the BT score (group 2b). Unadjusted values represent the association of injury types and GJH only

a = sex, age and sports participation significance, growth borderline significance

b = sex, age, growth and sports participation significance

c = sports participation significance

Sport participation was significantly associated with both the risk of traumatic and overuse knee injuries, as the more number of times the child participated in sport per week increased the risk of both types of injuries. For overuse knee injuries, a significant and positive association with sex, age and growth was also found in all analyses. Girls were more exposed to overuse injuries than boys and older children more exposed than younger children. Also, a large increase in height between the two tests rounds increased the risk of overuse knee injuries (Tables 4 and 5).

## Discussion

The main finding of the current study was firstly, that overuse injuries were the main knee injury type for both groups, with apophysitis being the most frequent diagnosis. Distortions and contusions were the most frequent traumatic injury diagnoses for both groups. Secondly, there was no significant increased risk of knee injuries in children with GJH, which questions the clinical relevance of recognising GJH as a risk factor for knee injuries in school children 9–14 years of age. A fluctuation in the status of GJH for a large number of children was observed between the two tests, with only one third of the children having a constant status of GJH.

In order to identify children with GJH throughout the study period, BT scores from both test rounds were matched. Consequently, only 36 children were classified with GJH on both test rounds, with the corresponding number of knee injuries for this group being low, indicating a risk of type 2 errors.

Some studies have reported an increased risk of traumatic knee injuries, including ACL injuries, for children and adolescents with GJH and/or knee hypermobility [10, 22, 33]. Opposing to the current school study, these studies were sports-specific, focusing on children or adolescents in soccer, basketball and netball; all being sports types involving high impact activities, which are known to increase the risk of traumatic injuries [8]. Due to the low number of children with GJH having traumatic injuries, the analyses were not stratified into sports type, which could be a confounder. In the current study, only three ACL injuries were registered, all in the control group, while distortions and contusions were the most frequent traumatic injuries for both groups.

No impact of GJH on knee injuries was seen in the current prospective, longitudinal cohort study involving children aged 9–14 years. In other prospective, longitudinal studies finding an increased risk for traumatic knee injuries, the study population was older and more mature, from the age of 14 upwards [22, 33]. The older and more mature children have larger body mass, are stronger and more powerful compared with younger children [34]. Also, older adolescents are typically more exposed to injuries due to more time spent in specialised sports with increased forces and loads, which may be one of the reasons why many ACL injuries occur in young athletes 15–25 years of age [8].

For both groups, overuse injuries were the most frequent injury type, with knee apophysitis, Mb. Sinding-Larsen-Johansson and Mb. Osgood-Schlatter, recognised as the most frequent diagnosis. Growth had a significant and exposing effect on these injuries with results being comparable to other studies [35, 36]. For the current age group, growth-related injuries are common [35]. Nevertheless, the extent of these injuries in school children has not been revealed until recently [31]. As apophysitis injuries are associated with growth, these injuries are self-limiting by nature [37], however, the short and long-term consequences of these injuries for sport participation and physical activity in general are not known. Traumatic injuries are known to cause both temporary and permanent disability for the individual with direct and indirect costs [38], which is why further studies following older adolescents with GJH in general populations as well as in sports-specific studies are suggested in order to identify if individuals with GJH are, or by increasing age will become, a high-risk population.

Generally, the prevalence of GJH in the current study was smaller than that in cross-sectional studies with comparable age, sex and ethnicity, especially for girls at 9–10 years of age (about 10 % smaller) and for boys at 9–10 years and 14 years of age (about 15 % and 10 % smaller) [16, 18, 39, 40], but similar to that in another large-scaled school-based, longitudinal cohort study [17]. Furthermore, in the current study, the prevalence of GJH within sex and age groups varied between the test rounds. For girls, the prevalence of GJH on the two test rounds gradually increased with age, and for boys it gradually decreased with age, in line with previous studies [19, 39–41]. The findings of the current study mainly relate to girls, which may hamper generalisability. However, since the prevalence of GJH among girls and women is generally higher, the current group may well be representative of GJH [42].

When GJH is described by prevalence within a child population, it may provide only a momentary picture for a group of children with GJH. Tracking the status of GJH for each child over time is another way to describe GJH within this population. For a large number of children, a fluctuation in the status of GJH was observed, whereas only 32 % of the children classified as GJH on the first test round were also classified with GJH on the second test round. This has never been reported and discussed before. Other longitudinal, school-based cohort studies have not specifically reported the tracking of status of GJH for each child in the cohort, but only 1 % (n = 18) of the children who had both lower limb pain at baseline and were classified with GJH at cut-point ≥6/9 at baseline, had a constant status of GJH at follow-ups [17]. Similarly, 1-2 % of the children with GJH and pain at baseline had a constant status of GJH at cut-point ≥6/9 and ≥5/9 at follow-up [43]. In the current study, a comparable percentage of 1.5 % of children with/without knee injuries had a constant status of GJH at cut-point ≥6/9.

The fluctuation in status of GJH for a given child could be explained by factors such as growth and particularly, a growth spurt. During rapid growth phases, a lengthening of bones occurs before muscles and tendons can stretch correspondingly [35], which may cause changes in joint mobility status. Frequent measurements every 3 months may therefore be implemented in future studies to capture the growth spurt phase specifically [44, 45]. In the current study, the prevalence of GJH for boys aged 13 was distinctly lower than for boys aged 12 on both test rounds, suggesting that during growth, boys seem to have a decrease in mobility; findings that were similar to two other studies [19, 41], but in contrast to another study [40]. The reverse was seen in girls aged 11 years on both test occasions, with the prevalence of GJH increasing at the age of 12, which is similar to another study with increasing prevalence of GJH at a mean age of 12.7 years [40]. As the current study found a potential and rather rapid fluctuation of the individual child’s status of GJH, it may be that a definitive classification of GJH might not be reported to the single child until post puberty.

Another explanation for the fluctuation in the status of GJH for an individual child could be a low inter-tester reproducibility. The BT was found to be reproducible (κ 0.64) for this child population and also comparable to the BT when performed a slightly different way, with no difference in the prevalence of GJH at cut-point ≥5/9 between methods [20]. The reproducibility of the total BT score is especially affected by tests not having clearly described and therefore not easily identified starting positions and clear endpoints, as can be the case with the knee and elbow tests [20], as described previously [21]. For the BT method applied to the current study, a substantial reproducibility was found for the knees (κ 0.62) [20].

However, positive predictive values (PPV) for BT for adults is suggested to be as low as 21 to 36 %, with a prevalence of 2-4 % and a sensitivity and specificity of the BT criteria of 93 %, although to date, no predictive validity study has been performed [15]. A low PPV questions the accuracy of the test in defining a condition, which means that classifying GJH may be a dilemma when examining the general population [15]. Therefore, low prevalence of GJH at cut point ≥5/9 must be taken into consideration, as PPV is directly proportional to the prevalence of the condition [46]. The theoretically assumed high NPV could have an impact on the results, biasing the results towards no impact of GJH on injury risk, hereby reducing the effect size, as there is a higher probability of false positive than false negative BT.

An alternative or supplementary test to the BT for knee hyperextension in standing could be to measure or verify knee hypermobility by goniometer measurements in supine lying, as applied in previous studies [22, 38] and/or measuring knee joint laxity, as these conditions seems to correlate in both adolescents and adults [24, 41]. In adults, GJH with simultaneous knee joint laxity was observed more frequently in ACL-injured persons [26], and correspondingly, GJH and knee joint laxity increased the risk of adult ACL injuries by 2.8 and 2.6 times, respectively [24]. In line with this, the children with GJH in the current study were stratified into groups with/without knee hypermobility, with increasing significance level for traumatic knee injuries in all analyses for those with knee hypermobility in addition to GJH. We classified GJH at a relatively high cut-point of at least 5/9 BT score points, but a requirement of also having to have simultaneous knee hypermobility may be stronger predictors for knee injuries than GJH only, as indicated in the current study and other studies [22, 24, 26, 47]. This hypothesis requires confirmation in future studies, but may explain why no association was found between knee injuries and GJH at lower cut-points not focusing on knee hypermobility [27, 48].

The weaknesses of the current study were the relatively low prevalence of children with GJH and knee injuries, which statistically did not allow for the a priori planned analyses with BT score at cut-points ≥6/9 (n = 16) and ≥7/9 (n = 1). Another weakness was that a large number of testers with different levels of clinical experience tested the children with the BT, which is a test with an unknown predictive validity. Although all testers were instructed and trained thoroughly in the standardised protocol, intra-tester or inter-tester reproducibility tests were not performed prior to each test round. An assurance of a high overall agreement for each tester may have eliminated any doubts of potential poor intra and inter-tester reproducibility. Still, an inter-tester reproducibility study for a previously similar protocol as applied in the current study was performed and moderate to substantial reproducibility was found [20].

The strengths of the current study are the longitudinal cohort study design and hence the possibility for frequent measurements of GJH, providing new knowledge about potential fluctuations of GJH over time for the individual child. Also, the frequent injury registration for traumatic as well as overuse injuries presents a broader and more valid representation of the extent of knee injuries in children aged 9–14 with GJH.

As growth spurts may affect both the type of injuries as well as the status of GJH, longitudinal studies with frequent examinations of GJH status are needed in order to evaluate if there is an association between GJH and knee injuries. When defining whether individuals with GJH are at risk of knee injuries, specific issues need to be considered. These considerations may include larger longitudinal studies, frequent measurements of GJH and growth due to possible changes in joint mobility, adding supplementary knee laxity tests, analysing injury risk also with higher cut-points of BT, as well as stratifying children and adolescents into specific sports types.

## Conclusions

In the current study, apophysitis, knee distortions and contusions were the most frequent knee disorders. No significantly increased risk of knee injuries was seen in children with GJH, which questions the clinical relevance of recognising GJH as a risk factor for knee injuries in children aged 9–14 years. A fluctuation in the individual child´s status of GJH between the two test rounds was observed for a large number of children, which suggests that inter- and intra-tester reproducibility of BT as well as growth may be considered important confounders to future studies of children and adolescents with GJH.

## Abbreviations

GJH: Generalised Joint Hypermobility
BT: Beighton Tests
ACL: Anterior Cruciate Ligament
SMS: Short Message Service
CHAMPS-study Denmark: The Childhood Health, Activity and Motor Performance School Study Denmark
